# Physicochemical Mechanics and Nonequilibrium Chemical Thermodynamics

**DOI:** 10.3390/e25091332

**Published:** 2023-09-14

**Authors:** Nikolai Meerovich Kocherginsky

**Affiliations:** Next-ChemX, University of Illinois at Urbana-Champaign, Hazelwood Dr., Champaign, IL 61820-7460, USA; n.kocherginsky@next-chemx.com or nm_koch@yahoo.com; Tel.: +1-217-3443250

**Keywords:** physicochemical mechanics, equilibrium and nonequilibrium thermodynamics, non-isolated systems, transport processes, mobility, second law of thermodynamics, thermodiffusion, colloid phenomena

## Abstract

Equilibrium thermodynamics answers the question, “by how much?” Nonequilibrium thermodynamics answers the question “how fast?” The physicochemical mechanics approach presented in this article answers both of these questions. It also gives equilibrium laws and expressions for all major transport coefficients and their relations, which was previously impossible. For example, Onsager’s reciprocal relations only tell us that symmetric transport coefficients are equal, and even for these, the value is often not known. Our new approach, applicable to non-isolated systems, leads to a new formulation of the second law of thermodynamics and agrees with entropy increase in spontaneous processes for isolated systems. Instead of entropy, it is based on a modified Lagrangian formulation which always increases during system evolution, even in the presence of external fields. This article will present numerous examples of physicochemical mechanics can be applied to various transport processes and their equilibriums, including thermodiffusion and different surface processes. It has been proven that the efficiency of a transport process with an actual steady-state flux (as opposed to a reversible process near equilibrium) is 50%. Finally, an analogy between physicochemical mechanics and some social processes is mentioned.

## 1. Introduction

A. Einstein in his Autobiographical Notes (1949) wrote: *“A theory is the more impressive, the greater the simplicity of its premises, the more diverse things it connects, and the wider its scope.”*

The total energy of an isolated system, even without equilibrium, stays constant, but entropy always increases during spontaneous internal processes, reaching its maximum at equilibrium. This statement is a modern way to formulate the second law of thermodynamics. This is why the modern presentation of nonequilibrium thermodynamics is based not on energy, which is conserved, but on entropy. An entropy increase gives information about the direction of a process, but it does not say how fast it happens. To answer this question, nonequilibrium linear thermodynamics assumes that the rates of transport processes are determined by the entropy production rate, ∂S/∂t. We will briefly remind the reader of the basics of nonequilibrium thermodynamics, and then we will describe a novel approach, which we will call physicochemical mechanics, and show its different applications and advantages. This new and simple approach is based on the Lagrangian, which includes both energy and entropy.

## 2. Basics of Nonequilibrium Thermodynamics

Changes in the movement of a mechanical body are described by Newton’s second law, according to which acceleration is proportional to a conservative mechanical force in Newton units, i.e., to the negative space derivative of potential energy *E*. The traditional description of system evolution in nonequilibrium thermodynamics is based on entropy and the second law of thermodynamics. The entropy of an isolated system increases during a spontaneous process, and, according to nonequilibrium thermodynamics, the rate of this process is proportional to the positive derivative of entropy versus a general coordinate. Instead of the traditional mechanical −∂E/∂X, it is always possible to introduce a partial derivative of entropy S$\left\{X_{k}\right\}$:

(1) $$F_{k}=\partial S/\partial X_{k}$$

where *E* is energy (*X*-space coordinate), $F_{k}$ is the generalized thermodynamic force, and $X_{k}$ is a generalized coordinate [1,2]. When one type of thermodynamic force is in equilibrium, $F_{k}$ is zero, and a deviation from zero shows how far the system is from equilibrium. $X_{k}$ does not necessarily have to be a Cartesian space coordinate and may be another variable. For example, if $X_{k}$ is the number of moles of one chemical component, $F_{k}$ is a partial molar entropy, ${\bar{s}}_{i}$.

In nonequilibrium thermodynamics, the rate at which a property $X_{k}$ changes is called the flux of this property, $J_{k}$, and

(2) $$J_{k}\equiv dX_{k}/dt.$$

It may be the flux of energy, entropy, etc., and it is not necessarily a vector flux in space.

To describe the evolution of a system (all fluxes), one must find all the thermodynamic forces, $F_{k}$, and this is carried out based on the rate of entropy production. The situation is straightforward in an isolated system, where *S* is an intensive function of only the internal energy, volume, and numbers of each component. If the system is not isolated, different fields change the total energy, and they can also lead to entropy changes. In this general case, the expression should include all the fluxes of the extensive variables, such as the number of molecules, volume, charge, energy, entropy, etc. As a result, using Equations (1) and (2), the entropy production is:

(3) $$J_{s}=\frac{dS}{dt}=\sum_{k}\frac{\partial S}{\partial X_{k}}\frac{dX_{k}}{dt}=\sum_{k}F_{k}J_{k}$$

For example, entropy production in chemical reactions is described by:

(4) $$\frac{dS_{r}}{dt}=-\sum_{i}\frac{\mu_{i}}{T}\frac{dc_{i}}{dt}$$

where $\mu_{i}$ is the chemical potential of the *i*-th component. For the concentration-driven molar flux of a noncharged species, its conjugated thermodynamic force is −$\mu_{i}$ divided by the absolute temperature, *T*.

In nonequilibrium thermodynamics, each flux of each different property (not only entropy) along the *x*-axis depends on all the thermodynamic forces, i.e., dropping vector notation,

(5) $$J_{k}=\sum_{j}L_{jk}F_{j}$$

Matrix coefficients, $L_{jk}$, are called phenomenological transport coefficients. They are usually not constants and depend on the local intensive parameters. This dependence should be found separately. For example, to describe energy-related entropy flux, the thermodynamic force is 1/temperature:

(6) $$\frac{dS}{dt}=\frac{1}{T}\frac{dE}{dt}$$

Often, heat transport is described by an experimental Fourier law: J=−κ∇T, where κ is thermal conductivity, and the thermodynamic force for heat transport is the difference of 1/T. It means that the phenomenological coefficient in this case is $\kappa T^{2}$, i.e., $J=\kappa T^{2}\nabla (1/T)$.

Though the values and even the signs of the nondiagonal matrix coefficients $L_{jk}$(k≠j) are often not known, when all fluxes in the system are described based on thermodynamic forces, the matrix of phenomenological coefficients is symmetrical, i.e.,

(7) $$L_{jk}=L_{kj}.$$

This celebrated statement is well-known as Onsager reciprocal relations. It was theoretically proven in 1931 based on the idea of reversibility near equilibrium [3,4]. In the presence of magnetic fields **B**, the situation is slightly different, as suggested in the 1945 Onsager-Casimir relation $L_{jk}(B)=L_{kj}(-B)$ [5]. The reciprocal relations decrease by half the number of all transport coefficients, which should be found to fully describe the system evolution. Only when a specific physical model is suggested can the nonequilibrium thermodynamics provide a clue as to what the sign and value of transport coefficients are. The problem is that without knowledge of all transport coefficients based only on linear thermodynamics, it is challenging to derive even classical equilibrium equations.

## 3. Basic Equation of Physicochemical Mechanics and Generalization of the Second Law of Thermodynamics

Note that according to Newton’s second law, the product of mass by acceleration is the total of all mechanical forces acting on a body. Thermodynamic forces are quite different from mechanical forces. If different $X_{k}$ have different units, different $F_{k}$ also have different units, not newtons or newtons/mol, and cannot be added. Thus, though linear thermodynamics gives several new fundamental results, it loses several fundamental properties of classical mechanics. Molecular dynamics and statistical mechanics are very helpful to describe transport, but the Fokker-Planck, Smoluchowski, and Langevin equations do not directly include entropy. On the other hand, information about any system near equilibrium is encoded in the entropy *S*, but this property, like temperature, does not exist in classical mechanics. Moreover, the temperature is usually kept constant, and it does not depend on space coordinates (isothermal processes). These are the reasons why even computer simulations did not give an adequate explanation of thermodiffusion [6].

There were recent attempts to improve the linear thermodynamics approach [7], but even before Onsager, there was a tendency to describe physical and chemical problems not based on a detailed molecular picture and statistical mechanics but on a general description, which does not require detailed knowledge of the molecular state. For example, in 1888, Joseph John Thomson published a book called “Applications of Dynamics in Physics and Chemistry” [8]. In this book, he tried to use Lagrange’s equation, generalized forces, and coordinates. Temperature was considered one of the generalized coordinates.

Slightly after Thomson, Pierre Duhem published two books in which he introduced the term chemical mechanics [9,10]. He also considered systems in terms of generalized forces and coordinates. Later, he introduced mechanical friction, i.e., dissipative processes, but dropped traditional inertial Lagrangian terms. As a result, his ideas were used not for transport but for chemical reactions and played a role in the emergence of twentieth-century thermodynamics for linear and even nonlinear processes.

The usual reasoning for linear Equation (5) is that any function can be presented as a power series, and in a narrow range near equilibrium, one can use the linear approximation. Later, a theoretical basis to describe far-from-equilibrium processes was further developed by Ilya Prigogine and his school [11]. In one of his publications, Prigogine wrote: “A more ambitious question is whether there is any thermodynamic function that, for dissipative systems, would at least in some sense play the role of the Lagrangian function in mechanics. Classical thermodynamics has solved the problem of the competition between randomness and organization in equilibrium situations. How then is it possible to extend these results to dissipative systems that are out of equilibrium either because they have not yet reached equilibrium or because the boundary conditions are such as to prevent them from going to equilibrium?” [12]. Though this problem attracted attention even after Prigogine, it is still not solved.

The Lagrangian is the difference between the kinetic and potential energies of the system. When the system is not isolated, in the presence of external fields, the negative gradient of potential energy *V_i_* gives macroscopic forces acting on particles “*i*”. This leads to directed transport and, in a condensed medium, energy dissipation due to friction. According to theoretical mechanics, the evolution of any multicomponent system with friction may be described by Lagrange’s equation with an additional Rayleigh’s dissipation function $\dot{Q}$ [13]:

(8) $$\frac{d}{dt}(\frac{\partial L}{\partial {\dot{x}}_{i}})-\frac{\partial L}{\partial x_{i}}+\frac{\partial \dot{Q}}{\partial {\dot{x}}_{i}}=0$$

Here L is the local Lagrangian of the system, and ${\dot{x}}_{i}$ is the velocity of the *i*-th particle along space coordinate *x*. Keeping the analogy to kinetic energy, Rayleigh suggested a dissipation function equal to half of the total dissipation per unit of time, i.e.,

(9) $$\dot{Q}=\frac{1}{2}\sum_{i}k_{i}{\dot{x}}_{i}^{2}$$

The partial derivative $\frac{\partial \dot{Q}}{\partial {\dot{x}}_{i}}$ is just the friction force with a minus sign:

(10) $$\frac{\partial \dot{Q}}{\partial {\dot{x}}_{i}}=k_{i}{\dot{x}}_{i}=-F_{i}$$

This is known as the Stokes law, which says that the friction force is proportional to velocity but has an opposite direction. Increasing velocity in condensed media will lead to increased friction forces. Very soon, it becomes equal to the total force acting in the opposite direction. Transport reaches a steady state, and now it is directed velocity and not acceleration (as it was in the Second Newton’s Law), which becomes proportional to the acting force [14]. When acceleration vanishes, $\frac{d}{dt}(\frac{\partial L}{\partial {\dot{x}}_{i}})=0$, and kinetic energy K does not depend on coordinates, i.e., $\frac{\partial K}{\partial x_{i}}=0$. Equation (8) becomes:

(11) $$-\frac{\partial E}{\partial x_{i}}=\frac{\partial \dot{Q}}{\partial {\dot{x}}_{i}}$$

Thus, the linear thermodynamic assumption that the system must be near equilibrium is not necessary anymore. Presented here, force-based interpretation does not need a more complicated function called Rayleighian, which is a total of two terms, one of them proportional to ${\dot{x}}_{i}^{2}$ and another to velocity ${\dot{x}}_{i}$. The advantage of this more complicated approach is that it leads to general Onsager’s variational principle and also to the Helmholtz principle of minimum dissipation energy, but it needs constant temperature and often gives only approximate solutions [15,16]. The relationship of Onsager reciprocity and fluctuation-dissipation relations with the principle of steepest entropy accent at given conservation constraints is discussed in [17].

Although temperature is not present in mechanics, it is a meaningful variable for a system with Brownian motion of many small particles. As we know, this motion does not stop even without any external force, and diffusion leads to directed transport from high to low concentration. Force-based interpretation in physicochemical mechanics easily leads to the introduction of entropic and thermal forces. It is determined by the entropy gradient at a constant temperature kept by the thermal reservoir and is important for diffusion. This entropic force can be presented as $F_{s}=T\frac{\partial S_{i}}{\partial x_{}}$. There is no minus sign, and this force is directed towards the increase of entropy, as it should be according to the Second Law of thermodynamics. Another force, determined by the gradient of temperature at constant entropy, is important for thermodiffusion, $F_{T}=S_{i}\frac{\partial T}{\partial x_{}}$. In addition to the directed drift induced by external fields, dissipation influences the thermal motion of molecules and often leads to a temperature increase. Note that both $F_{s}$ and $F_{T}$ have the same units as mechanical forces.

Assuming that potential energy is an additive function of its components (ideal, noninteracting components), and both partial (for given species *i*) potential energy and partial entropy are proportional to the number of molecules $N_{i}$, we can use partial molar entropy ${\bar{s}}_{i}$ and partial potential energy ${\bar{V}}_{i}$. Finally, for directed molecular velocity, we have:

(12) $$v_{i}=\frac{N_{i}}{k_{i}}[\frac{\partial ({\bar{s}}_{i}T)}{\partial x_{i}}-\frac{\partial {\bar{V}}_{i}}{\partial x_{i}}]$$

Thus, we derived the general equation, saying that transport is determined by the balance of two general driving factors. One is the increase in local heat $S_{i}T$, which is important for small particles. Another term is the gradient of potential energy with a minus sign. The function in square brackets is a modified Lagrangian, and it always increases in system evolution even in the presence of external fields. In addition, excessive heat is transferred to the surroundings. Of course, for an isolated system without external fields and at constant temperature, this new formulation is reduced to the classical Second Law of thermodynamics.

Classical Newtonian mechanics dealt with the movement of a body driven by different external forces, but it did not consider Brownian motion or thermal collisions of molecules. The central principle was a decrease in potential energy. When the movement of much larger bodies than molecules is considered, thermal motion leading to changes in entropy does not play an essential role, and the system simply evolves towards the decrease of its potential energy. Classical thermodynamics started with a description of heat engines, and heat is closely related to molecular movement. The primary principle in this case is the increase in entropy for isolated systems. Leading to ordering, field-driven molecular drift is balanced by counteracting and leading to disordered *ST*-related movement, and the whole process reaches the steady state or even stops because of friction [14]. Thus, now we have a unified description of both molecular and macrosystems. For nonhomogeneous systems influenced by external fields and not isolated systems, this description should be used instead of the Second Law, and it also explains the possibility of ordering from chaos.

## 4. General Equation for Chemical Flux

To illustrate different applications and consequences of our approach, we will need the molar flux $J_{i}$ per unit area (moles/area/time), which is $J_{i}=cv_{i}$. We also introduce mobility $U_{i}$, like A. Einstein did for diffusion [18]. After that,

(13) $$J_{i}=U_{i}c_{i}[\frac{\partial ({\bar{s}}_{i}T)}{\partial x_{i}}-\frac{\partial {\bar{V}}_{i}}{\partial x_{i}}].$$

To have a more familiar expression for flux, we further introduce a function that is similar to the Gibbs free energy *G* of the homogeneous system. The difference is that now it is more general, depends on coordinates, and is sensitive to external fields:

(14) $$G_{g}(x)=\sum_{i}({\bar{E}}_{ini}+\sum_{j}k_{ji}\psi_{j}+{\bar{\upsilon }}_{i}P-{\bar{s}}_{i}T)N_{i}$$

${\bar{E}}_{ini}$ is the molar internal chemical energy of the *i*-th component. It is determined by the molecular structure of all components and their mutual interactions. Coefficients $k_{ji}$ are partial molar properties conjugate to their related fields, such as molar charge, molar weight, etc. All products of local potentials and conjugate partial molar properties have units of molar energy (Joule/mole). The three first terms in brackets are total molar chemical and potential energy, and the third term ${\bar{s}}_{i}T$ is molar thermal kinetic energy. Thus, $G_{g}$ is an analog of mechanical Lagrangian with a minus sign.

An analog of chemical potential, which we call physicochemical potential, for each component is:

(15) $$\mu_{gi}(x)={\left(\frac{\partial G_{g}}{\partial N_{i}}\right)}_{P,T,N_{j\neq i},\psi_{k}}=\mu_{i}^{0}+\sum_{j+1}k_{ji}\psi_{j}-{\bar{s}}_{i}T$$

The summation here includes the term external pressure. The field potentials $\psi_{j}$, pressure, temperature, and even the analog of the standard chemical potential $\mu_{i}^{0}$ in nonhomogeneous systems are functions of coordinate. This variable is especially important for asymmetric membranes. For example, even nonionic surfactants, when added from one side of a biomimetic membrane, change $\mu_{i}^{0}(x)$ into ions and lead to transient changes in transmembrane electric potential [19].

Evidently,

(16) $$-\frac{d\mu_{gi}}{dx}=\frac{d({\bar{s}}_{i}T-\mu_{i}^{0}-\sum_{j+1}k_{ji}\psi_{j})}{dx},$$

Now each component of the total gradient has the same units of molar force (Newton/mole). Due to this, they may be added, which is different from thermodynamic forces with different units. Without chemical reactions and external fields, we have simple $-\frac{d\mu_{gi}}{dx}=\frac{d({\bar{s}}_{i}T)}{dx}$. This equation for each component describes partial free energy dissipation into heat. If the physicochemical potential $\mu_{gi}$ of a component is independent of other components, in spontaneous processes it always decreases, even if the system is influenced by external fields. Substitution yields the previously proposed relationship of physicochemical potential and flux [20]:

(17) $$J_{i}=-U_{i}c_{i}\frac{\partial \mu_{gi}}{\partial x_{i}}$$

This flux of each component is proportional to the concentration’s total driving force, i.e., the local gradient of the physicochemical potential. While the molar flux of component *i* may be driven by any combination of different forces, it is characterized by a single mobility *U_i_*, which has the units of velocity/molar force.

Instead of common isobaric-isothermal processes, it is possible to simultaneously apply a pressure gradient, temperature gradient, and electric field and observe chemical transport. For non-isolated systems, instead of the chemical potential $\mu_{i}$, it is the physicochemical potential $\mu_{gi}$ that becomes constant in equilibrium. Physicochemical potential $\mu_{gi}$ includes the product of molar entropy and temperature with the minus sign. Other terms are positive products of molar properties driven by conservative driving factors, such as molar volume and pressure. As a result, when partial potential energy goes down and/or molar entropy goes up in any spontaneous transport process, $\mu_{gi}$ still decreases. Dissipation stops in equilibrium when all molecular forces balance each other. The Lagrangian of the system (difference of kinetic and potential energy) reaches its maximum and becomes time- and coordinate-independent, reminding us of the situation with the Onsager variational principle [21].

Classical thermodynamic entropy does not depend on external fields, but if properly used together with the additional fields, entropy-based and Lagrangian-based approaches, i.e., nonequilibrium thermodynamics and mechanics for nonisolated systems, are closely related. It is known that the thermodynamic entropy of a monocomponent ideal solution may be presented as:

(18) $${\bar{s}}_{i}=s_{0}+{\bar{C}}_{\upsilon }\ln T-R\ln c_{i}$$

*R* is the universal gas constant [22]. This equation may be substituted into Equation (16), and after regrouping together with $\mu_{i}^{0}+{\bar{\upsilon }}_{i}P$, we have an expression for traditional chemical potential as a function of concentration. Thus, further description may be presented either in terms of physicochemical and chemical potential, which was the case in our previous papers [23,24], or in terms of physicochemical potential and entropy, which is the case here.

The major purpose of physicochemical mechanics is to describe transport processes. Without chemical reactions, when internal energy is constant, the complete differential $G_{g}$ may be presented as:

(19) $$dG_{g}=\sum_{i}\mu_{fi}dN_{i}+\sum_{i}N_{i}d\mu_{fi}+PdV+VdP-TdS-SdT.$$

$\mu_{fi}$ reflects total force potential. For transport, we need only a part of Equation (19) with differentials of intensive variables.

(20) $$dG_{g}=\sum_{i}N_{i}d\mu_{fi}+VdP-SdT.$$

Together with the previous equation, this means that:

(21) $$\sum_{i}\mu_{fi}dN_{i}+PdV-TdS=0.$$

Note that the role of natural variables and their conjugated molar properties in transport changes to the opposite in comparison to chemical processes. There is no contradiction of this and well-known for chemical reactions Gibbs-Duhem equation $\sum_{i}N_{i}d\mu_{i}-VdP+SdT=0$ because the first is the result of vectorial transport and the second describes the equilibrium of scalar chemical reactions [22].

## 5. Diffusion, Other Transport Laws, and Their Equilibrium

For transport of one component using the relation of $\mu_{gi}$ and ${\bar{s}}_{i}T$ for homogeneous isolated systems at constant temperature, we have:

(22) $$J_{i}=-U_{i}RTc_{i}\frac{\partial \ln c_{i}}{\partial x}=-D_{i}\frac{\partial c_{i}}{\partial x}.$$

Thus, this simplest example leads to the first Fick’s law and the Einstein-Fokker relation of mobility and diffusion coefficient, $D_{i}=RTU_{i}$. Diffusion coefficients *D* of ions in water at room temperature are near 10^−9^ m^2^/s, which corresponds to mobility $U\approx 4\times 10^{-13}\frac{m}{s}\cdot \frac{mol}{N}$.

Another important driving factor is the gradient of an electric potential $\psi_{e}$. If all other gradients, including the concentration gradient, are zeros, we have:

(23) $$\frac{\partial \mu_{gi}}{\partial x}=\bar{q}\frac{\partial \psi_{e}}{\partial x}$$

(24) $$J_{i}=-z_{i}FU_{i}c_{i}\frac{\partial \psi_{e}}{\partial x}$$

*F* here is the Faraday number and $z_{i}$ is an elementary ionic charge. Faraday’s law for electrophoresis states that the ion migration is proportional to the applied field and molar charge $\bar{q}$. If concentration of dissolved 1:1 salt is 10^3^ mol/m^3^ (1 mole/L), using the value for $U_{i}$_,_ the specific electrophoretic mobility induced by both monovalent ions should be $∼8\times 10^{-5}mol/s\cdot m\cdot V$, which agrees with the experiments. Taking one term in physicochemical potential after another, we will get other transport laws and the values of related transport coefficients.

This analysis easily leads to the well-known equilibrium laws. For example, at equilibrium, concentration-driven and electric field-driven fluxes should balance one another. After integration, this leads to the Nernst law, describing transmembrane electric potentials $\Delta \psi_{e}$ as a function of two concentrations $c_{i1}$ and $c_{i2}$ separated by the membrane solutions:

(25) $$-U_{i}RT\frac{\partial c_{i}}{\partial x}=z_{i}FU_{i}c_{i}\partial \psi_{e}/\partial x$$

(26) $$\Delta \psi_{e}=-\frac{RT}{z_{i}F}\ln\frac{c_{i2}}{c_{i1}}$$

Similar analysis may be performed for the balance of concentration and the gradients of any other external potential fields, such as gravity or pressure gradients (barodiffusion). Even simpler analysis may be performed for an equilibrium of the gradients of two fields, for example, for piezoelectric materials with pressure and electric field as driving factors. For nonideal or nonhomogeneous solutions, it is possible to add the gradients of the standard chemical potential or of the logarithm of the activity coefficient, which may be considered internal fields. The result does not depend on mobility in all these cases, as it does in equilibrium thermodynamics.

## 6. Colloid Materials

The above analysis is valid for cases when the coefficients $k_{ji}$, conjugate to their related variables, such as molar volume, molar charge, and molar weight, stay constant. In the cases of colloid phenomena and thermodiffusion, the situation is more complex. Below, we consider both of these cases and also mention a few aspects of mechanochemistry to show application of $k_{\mu }$ in mechanical stress analysis.

The possibility of using conventional non-equilibrium thermodynamics for interfaces is not obvious, which was recognized by I. Prigogine and other authors [25,26]. The reason may be that in colloid materials, there are not one but three possible driving factors. These are surface tension σ, molar surface area $\bar{a}$, and curvature. It should also be clear that to keep classic equilibrium laws valid, we must use the same mobility for surface and volume transport. For example, if all other driving factors are constant, we postulate that the rate of lateral transfer of an adsorbed component due to the gradient of surface tension is:

(27) $$J=-Uc\bar{a}\frac{\partial \sigma }{\partial x}$$

Here c is the local concentration near the surface. $\bar{a}$ is constant and σ changes as a function of x. Now it is easy to describe the equilibrium of processes driven by surface tension and any other driving factor, such as concentration, pressure, and electric field. In the first case, together with Fick’s law, if molar surface area $\bar{a}$ is constant, after integration we have:

(28) $$\ln\frac{c_{2}}{c_{1}}=-\frac{\bar{a}\Delta \sigma }{RT}$$

This equation can be rewritten in a differential form, known as the Gibbs adsorption isotherm:

(29) $$-\frac{cd\sigma }{RTdc}=\frac{1}{\bar{a}}=\Gamma$$

Here Γ is the surface concentration.

The balance of surface tension and electric potential gives the Lippmann (1880) equation, used to analyze an electric double layer and electrocapillary maximums [27]:

(30) $$\frac{d\sigma }{d\psi }=-\frac{\bar{q}}{\bar{a}}$$

Changes in applied electric potential lead to changes in surface tension, and the negative slope reaches its zero maximum when the surface charge is zero. This zero-charge potential is an essential characteristic of electrode kinetics, especially for polarography with dropping mercury electrodes.

For a pair of surface tension and pressure as driving factors at equilibrium:

(31) $$\frac{d\sigma }{dP}=-\frac{\bar{\upsilon }}{\bar{a}}=-\bar{\upsilon }\;\Gamma$$

For a sphere, it is reduced to the Young-Laplace equation:

(32) $$\Delta P=-\frac{\bar{a}\sigma }{\bar{\upsilon }}=-\frac{2\sigma }{r}$$

In the case of the Marangoni effect, the gradient of surface tension leads to pressure gradients and hydrodynamic volume flow. It is important that mobility stays the same for volume and surface. This is not surprising because, according to Stokes law, it is proportional to 1/viscosity, which determines hydrodynamic fluxes. Simultaneously, this shows potential applications to viscose flow and capillary hydrodynamics and may be compared with the results based on the Onsager variational principle [17,21].

For a sphere, it is the ratio 2σ/r that plays the role of a driving factor. Its small changes are:

(33) $$\delta (2\sigma /r)=\frac{2}{r}\delta \sigma -\frac{2\sigma }{r^{2}}\delta r$$

If *r* is changing, after integration, the balance of the second term with electric forces gives:

(34) $$\psi_{2}-\psi_{1}=-\frac{2\sigma \bar{\upsilon }}{zF}(\frac{1}{r_{2}}-\frac{1}{r_{1}})$$

The size or even curvature of a colloid or nanoparticle particle is a new possible variable that is not important for bulk processes. Changes in electric potential with size play a crucial role in polarography with dropping mercury electrodes, colloid stability, nanoparticle electroseparation, and electrocapillary effects. In addition, a species has higher energy on the surface with a higher curvature and will move to the flat area. The flux due to the changes in *r* should be proportional to $-Uc\bar{\upsilon }\frac{2\sigma }{r^{2}}$. In equilibrium, this flux must be counterbalanced by a concentration-driven flux:

(35) $$RT\frac{dc}{dr}=\frac{2\sigma \bar{\upsilon }c}{r^{2}}$$

Integration from *r* to ∞ using partial pressure for vapor instead of concentration gives the Kelvin equation for partial vapor pressure above the curved surface:

(36) $$RT\ln\frac{p_{r}}{p_{\infty }}=\frac{2\sigma \bar{\upsilon }}{r}$$

If in the process $\bar{a}$ changes and σ is kept constant, the balance of surface effects and electrical forces is described by:

(37) $$\frac{d\psi }{d\bar{a}}=-\frac{\sigma }{zF}$$

The mechanical properties of the material can be used to calculate the surface tension of a solid phase. In the reversed phenomenon, adsorption of surface-active substances and their subsequent penetration decrease σ on the surfaces of solid granules inside the solid. This process results in increasing stress, plastic deformation, and even spontaneous destruction and dispersion. For example, it is possible to break a Zn metal plate with almost 1 mm of thickness by hand or break a wedding gold ring simply by covering it with micrometer-thick films of Hg. In these phenomena, known as Rehbinder’s effect, an adsorption-induced decrease of surface tension leads to smaller inter-particle interactions and, finally, reduces the material’s mechanical strength due to preexisting minor cracks.

Young’s module *Y* gives the relationship between elastic stress $\pi_{el}$ and strain *l*: $d\pi_{el}=Ydl/l$. If temperature changes, neglecting entropy changes, we may write:

(38) $$\frac{d\pi_{el}}{dT}=\frac{\bar{s}}{\bar{\upsilon }}$$

Entropy has the term –*Rlnc*. If *c* is high enough, tensile stress and *Y* decrease at a higher temperature, but the mechanical strength (difference between elastic stress limit and internal stress) increases. This effect is usually observed in metals and ceramics.

Besides the thermodynamic effects, mechanical stress can also affect rates of transport between granules and the formation of dislocations. For example, the rate of hydrogen transport in a palladium plate increases when the direction of stress is the same and decreases when it is opposite to the direction of diffusion. A well-known example of interacting chemical, transport, and mechanical processes is biological muscle contraction, which is sensitive to lipid stress and mechanosensitive voltage-gated channels. Stress may be strong enough to lead to the mechanical rupture of chemical bonds and the formation of free radicals.

## 7. Thermodiffusion

All transport processes we discussed are conducted at constant temperature, though the temperature gradient may also lead to mass transport. This process is called the Soret effect or thermodiffusion. Thermodiffusion and related processes are the last major molecular transport phenomena that still lack a simple physical explanation [28,29]. The major reason now is clear: molar entropy in the product ${\bar{s}}_{i}T$ is not only a function of coordinate but also a function of temperature. Usually, the total process is described based on an analogy to Fick’s law:

(39) $$J=-D\frac{dc}{dx}-S_{T}Dc\frac{dT}{dx}$$

The experimental Soret coefficient *S_T_* has the dimension *1/K*.

According to the approach presented here and Equation (18), the flux driven by the gradient ${\bar{s}}_{i}T$ is:

(40) $$J_{\bar{s}T}=Uc\frac{d}{dx}(\bar{s}T)=Uc({\bar{C}}_{\upsilon }\ln T-R\ln c+s_{0})\frac{dT}{dx}+UcT(\frac{{\bar{C}}_{\upsilon }}{T}\frac{dT}{dx}-\frac{R}{c}\frac{dc}{dx}+\frac{ds_{0}}{dx})$$

If we use the approximation that $s_{0}$ is not temperature-dependent, the expression is simpler:

(41) $$J_{\bar{s}T}=-URT\frac{dc}{dx}+Uc(\bar{s}+{\bar{C}}_{\upsilon })\frac{dT}{dx}$$

Again, we have recovered Fick’s law and the Fokker-Einstein relation D=RTU. In addition, we have a term with an opposite sign, which gives temperature-driven flux. Now the Soret coefficient is:

(42) $$S_{T}=-\frac{{\bar{C}}_{\upsilon }+\bar{s}}{R}\frac{1}{T}$$

If we further neglect ${\bar{C}}_{\upsilon }$, an expression for $S_{T}$ is reduced to a simple negative ratio of molar entropy and *RT* [30]. At high concentrations and low temperatures $S_{T}>0$, transport is directed from hot to cold. In the opposite situation (low c, high *T*) $S_{T}<0$, transport is from cold to hot. When concentration is increased, molar entropy goes down, which may lead to changes in the sign of the Soret coefficient from negative to positive and, as observed in experiments, changes in the flux direction.

Only if the system is isolated (no external fields) and the temperature is constant will transport be towards the increase of molar entropy, leading to the Second Law of thermodynamics. The final state with maximum entropy is characterized by constant concentration throughout the system. This is not true for systems influenced by external fields, not isolated systems, which could evolve in active transport to a more ordered state with decreased entropy. Further, it is easy to add the term electric potential, which will explain thermoelectric phenomena. Thus, we can explain many nontrivial experimental effects in thermodiffusion and related processes.

## 8. Onsager’s Reciprocal Relations

If the same transported species carries different properties, to calculate the fluxes of these properties, such as volumetric flux or electric current, we must multiply Equation (17) by the corresponding conjugated properties, such as molar volume or molar charge. For example, consider the charges flowing in the *x*-direction in a conductor of cross-section *A* and length *L*. In this case, electric current is I=AzFcv (coulomb/s), and voltage is related to the electric field as $E_{x}=\Delta \psi_{e}/L$. Making these substitutions on the left and right-hand sides of the equation $v_{x}=zFUE_{x}$, we obtain:

(43) $$IR=\Delta \psi_{e}$$

This is Ohm’s law, with the resistance proportional to the length and inversely proportional to the area *A*, *R* = *L*/(*z*^2^*F*^2^*UAc*).

Now we are ready to go back to the beginning of this paper and discuss the Onsager’s reciprocal relations. In this case, we need several fluxes carried simultaneously by several forces. For simultaneous mass and charge transport, we have two fluxes: molar flux and electric current, driven by two forces:

(44) $$\begin{array}{l} J=Uc[-\frac{d\mu }{dx}-\frac{zFd\psi }{dx}]=L_{11}\frac{d\mu }{dx}+L_{12}\frac{d\psi }{dx}\\ I=UczF[-\frac{d\mu }{dx}-\frac{zFd\psi }{dx}]=L_{21}\frac{d\mu }{dx}+L_{22}\frac{d\psi }{dx} \end{array}$$

and evidently $L_{12}=L_{21}=-UczF$. Based on this example, it is easy to see that similar manipulation with other partial molar coefficients leads to $L_{ij}=L_{ji}$ in the general case. Moreover, using the same U and usually known different molecular properties, we can now calculate the values of all and not only diagonal transport coefficients, which was impossible with Onsager’s approach.

If we use $+d(\bar{s}T)/dx$ as a molar force, the sign of the reciprocal relation will change to minus. A similar situation was mentioned above for Onsager-Casimir relations in magnetic phenomena, and now it gets a simple explanation: both magnetic forces and entropic forces are not conservative and increase with velocity.

Note that Onsager’s assumption regarding microreversibility near equilibrium is not necessary in this analysis. It is also clear that simple reciprocal relations are not valid for multicomponent diffusion, where different independent components have different mobility coefficients. A new type of reciprocal relation for multicomponent diffusion was suggested based on physicochemical mechanics in [23].

## 9. Dissipation and Thermodynamic Efficiency

The thermodynamic efficiency of working between two temperatures in heat engines is described by Carnot’s cycle [28]. Physicochemical mechanics allows the derivation of thermodynamic efficiency for isothermal non-equilibrium processes. Driven by external forces, the transport of a component *i* is characterized by useful work and is given by an integral:

(45) $$W_{i}=\int_{0}^{x}\frac{d\mu_{gi}}{dx}dx$$

In its turn, energy dissipation because of a frictional force $F_{fi}$ is:

(46) $${\dot{Q}}_{i}=\int_{0}^{x}F_{fi}dx=-\int_{0}^{x}\frac{k_{i}}{N_{i}}v_{i}dx=-\int_{0}^{x}\frac{k_{i}}{N_{i}}\frac{J_{xi}}{c_{i}}dx=\int_{0}^{x}\frac{\partial \mu_{gi}}{\partial x}dx,$$

where we used the relationship between Einstein’s molar mobility and friction coefficient $U_{i}\equiv N_{i}/k_{i}$. Thus, useful work is precisely equal to the energy lost due to dissipation in this elementary process, meaning that the efficiency of a process with actual flux (as opposed to a reversible process near equilibrium) is exactly 50%.

## 10. Summary

Presented here and related to the theoretical mechanics approach, which we call physicochemical mechanics, leads to the systematic derivation of all major chemical transport laws on a molecular level. It also gives the value and relationship of all transport coefficients, which are based on partial molar properties conjugated to different driving factors and on molecular mobility. If we know these properties, we can predict what the rate of transport will be, which was impossible in Onsager’s thermodynamics. For several simultaneous transport processes carried by one species, we easily get the Onsager’s reciprocal relations, but we also show their limitations for multicomponent systems.

For nonisolated and interacting systems with external fields, we suggest a new formulation of the Second Law of Thermodynamics based on a Lagrangian instead of entropy. In other words, all molecular transport processes (system evolution) are determined by forces due to the balance of potential energy and related to the Brownian motion of thermal kinetic energy.

The balance of two driving forces leads to related classical equilibrium equations. The table of all major molecular transport laws was previously published based on seven major driving factors, including those for colloid science and thermodiffusion [20]. Further, it is possible to add new driving factors, such as light, dipole interactions, important for solid bodies, mechanical deformations, etc., and not only predict new phenomena but even how fast they will be. In the future, it will also be possible to extend this approach to hydrodynamic processes.

Physicochemical mechanics may be used not only for chemical transport but also to describe the kinetics of elementary irreversible and reversible chemical reactions far from equilibrium. In this case, instead of the space coordinate x, we can use the reaction coordinate. The integral form of the transport equation leads to exponential expressions for rate constants, and it is not necessary to introduce additional assumptions regarding a transitional state [24].

Finally, we can make a few general comments that may be useful for the social sciences. More general social processes are always accompanied by interactions, collisions, and frictions, and an analog of mobility could characterize how favorable the media is for progress. For example, the social striving for liberty may be considered a manifestation of the Second Law of Thermodynamics [31]. The rate of progress may be characterized by an analog of flux, which should have different units for different processes. Still, it should be proportional to the product of mobility and total driving force. This total force is the difference between ordering permanent driving factors and chaotic migration in the opposite direction. It seems reasonable to assume that modern society is a rather ordered system that works like a machine, producing something useful, which may be called work. Otherwise, at least because of anarchy, no work will be carried out. Certainly, some order and constraints, like laws and country borders, are necessary to do the work. Its purpose is progress, which is preferably achieved via evolution, via smooth transitions from one stable state to another. With time, too much order and dictatorship will deprive the system of mobility and efficiency. One of the ways to achieve efficient evolution is to change the media, to provide more mobility and degrees of freedom, and to have strong positive stimulative forces (“increase of Lagrangian”) while remaining sustainable, i.e., stable enough to withstand natural and political catastrophes driven by external forces and to avoid phase transitions, i.e., wars and revolutions. Finally, a more detailed description of physicochemical mechanics and its applications may be found in the book [32].

## Data Availability

No new data were created in this review.
